# The impact of peer effects on adolescents' physical exercise: a meta-synthesis study

**DOI:** 10.3389/fpsyg.2025.1748992

**Published:** 2026-01-29

**Authors:** Kai Chen, Cheng Chen, Jincheng Yang, Zhongdong Li

**Affiliations:** 1 Basic Teaching Department, Yantai Health Vocational College, Yantai, China; 2 Graduate School, Harbin Sport University, Harbin, China

**Keywords:** peer effects/partner engagement, phenomenology, qualitative research, mixed methods research, grounded theory, youth/children/sports/exercise/participation

## Abstract

This study employed a meta-synthesis approach to systematically review qualitative and mixed-methods research on the influence of peer effects on adolescents' physical exercise behavior, aiming to provide a theoretical basis and practical pathways for promoting adolescent sports health. Through literature screening and quality assessment, 28 articles were included, yielding 45 research findings. These were categorized into 12 subcategories and synthesized into four main integrated findings: Peer Support and Interaction; Behavioral Modeling and Atmosphere; Intrinsic Motivation and Identity; and Educational Interventions and Risk Management. The study found that peer effects significantly influence adolescents' participation and persistence in physical exercise through multiple pathways, including emotional support, behavioral modeling, and motivational atmosphere. However, potential risks such as dependency and group heterogeneity exist. Future educational practices should emphasize differentiated guidance, establish scientific peer support systems, effectively mitigate potential risks while leveraging positive effects, and promote the long-term healthy development of adolescents' physical activity behaviors.

## 1 Introduction

Adolescence constitutes a critical phase for individual physical, cognitive, and psychosocial development. Regular physical exercise during this period not only enhances cardiopulmonary function, muscular strength, and metabolic health, but also contributes to psychological wellbeing, stress resilience, and social adaptation. Extensive evidence indicates that active adolescents exhibit lower levels of anxiety and depression, higher self-esteem, and better academic performance (Zhang et al., 2022). Despite these recognized benefits, recent national surveys reveal a concerning trend: physical activity levels among Chinese adolescents remain substantially below international recommendations, with a high prevalence of sedentary behaviors and rising rates of obesity, myopia, and metabolic syndromes (Wang et al., 2023). This “inactivity crisis” underscores an urgent public health priority and calls for deeper investigation into the factors that can effectively promote sustainable exercise engagement in youth (Sacerdote, 2011).

Among the various psychosocial determinants of adolescent behavior, peer influence emerges as one of the most salient. Rooted in social network theory, peer effects refer to the process through which individuals' attitudes, emotions, and behaviors are shaped by the norms, expectations, and actions of their peer group. During adolescence, peers gradually supplant family as the primary social reference, wielding considerable influence over lifestyle choices, identity formation, and daily routines. In the domain of physical activity, existing research suggests that peer support, observational learning, and group norms can significantly boost adolescents' motivation, participation frequency, and skill acquisition (Brown and Larson, 2009). Conversely, peer rejection, social comparison pressure, or negative group climates may serve as barriers to consistent involvement. Nevertheless, while a growing body of literature acknowledges the relevance of peer effects, most studies remain correlational or fragmented in scope (Smith et al., 2015). There is a notable scarcity of integrative, mechanism-focused reviews—particularly within the Chinese educational and cultural context—that systematically synthesize how peer dynamics operate across emotional, behavioral, and cognitive dimensions (Jiang and Zhang, 2021). Moreover, prior work seldom addresses the potential dual nature of peer influence, which may entail both facilitative and risk-laden pathways.

Therefore, this study employs a meta-synthesis methodology to systematically review and integrate qualitative and mixed-methods evidence on peer effects in adolescent physical exercise (Li et al., 2024a). By synthesizing findings across different settings and populations, we aim to achieve three objectives: (1) to elucidate the multidimensional manifestations and underlying mechanisms through which peers influence exercise behavior; (2) to identify contextual and individual factors that moderate these effects; and (3) to derive evidence-informed implications for designing school-based, family-involved, and community-supported interventions. Ultimately, this work seeks to provide a coherent theoretical framework and practical guidance for leveraging positive peer dynamics while mitigating associated risks, thereby fostering sustained, health-promoting physical activity patterns among adolescents.

## 2 Research methods

### 2.1 Search strategy

A combination of subject headings and free-text terms was used to search databases including PubMed, Embase, Cochrane Library, Web of Science, CNKI, Wanfang, and VIP for qualitative and mixed-methods studies on peer effects in adolescent physical exercise or physical education classes. Key English search terms included “youth/children/sports/exercise/participation,” “peer effects/partner influence/partner engagement,” “Qualitative research, mixed methods research, grounded theory, phenomenology, narrative research, action research, case study,” etc. The search period spanned from database inception to November 2025.

### 2.2 Literature inclusion and exclusion criteria

To systematically synthesize existing qualitative research and deeply explore the specific manifestations and mechanisms of peer effects in adolescent physical exercise, this study established clear literature screening criteria. Inclusion criteria were as follows: (1) Participants: Students in the adolescent stage; (2) Phenomenon of Interest: Experiences, feelings, and perceptions of being influenced by peers (e.g., support, modeling, norms, rejection) during participation in physical exercise; (3) Context: Physical exercise settings in schools, communities, or daily life; (4) Study Type: Qualitative research or mixed-methods studies containing extractable qualitative research components.

Exclusion criteria were: (1) Systematic reviews, overviews, or commentary articles; (2) Literature not in Chinese or English; (3) Literature for which full text was unavailable or data could not be extracted.

### 2.3 Literature search and screening process

This study systematically searched Chinese and English databases. First, all retrieved records were imported into EndNote X9 reference management software to remove duplicates. Subsequently, two researchers independently screened the titles and abstracts of the records according to the inclusion and exclusion criteria. For records that passed the initial screening, full texts were obtained and carefully reviewed for final inclusion decisions. Any disagreements during the screening process were resolved through discussion or by consulting a third researcher.

### 2.4 Data extraction and quality assessment

The extracted data primarily included: author and publication year, study location/country, research methods, participant characteristics (e.g., age, gender), specific physical exercise context, core themes related to peer effects (e.g., types of peer support, pressure from group norms, experiences of social exclusion), and main research findings. The Critical Appraisal Skills Programme (CASP) checklist from the Oxford Center for Evidence-Based Medicine was used to assess the quality of included studies. This checklist consists of 10 questions, each rated as “Yes,” “No,” or “Unclear.” Based on the assessment results, the quality of included studies was classified into grades A, B, or C: meeting all criteria was graded A, partially meeting criteria was graded B, and meeting none was graded C. Studies graded B and above were ultimately included.

## 3 Results

### 3.1 Search results

**Figure 1:**
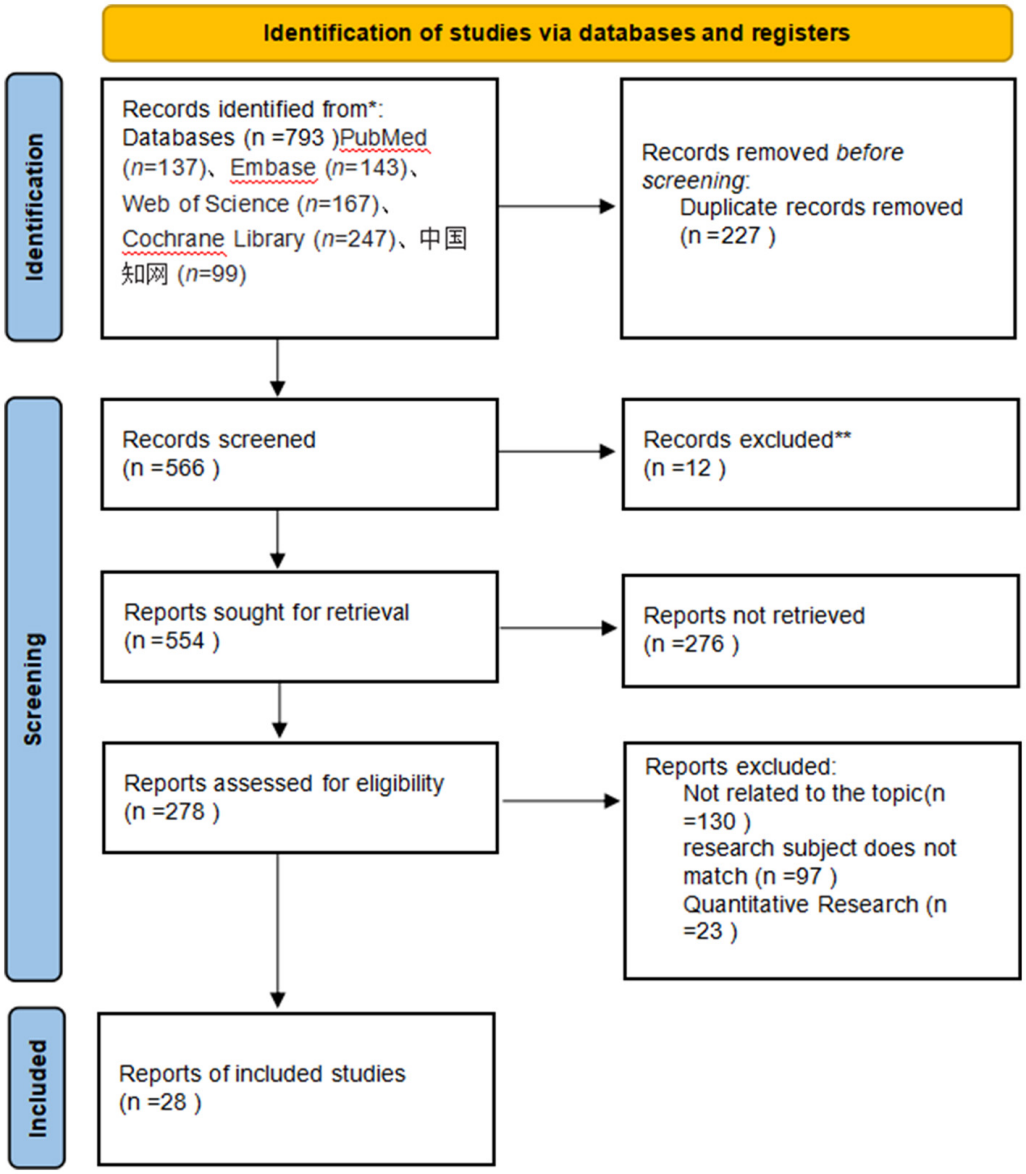
Literature screening flowchart.

The initial search identified 793 relevant records. After removing duplicates, 566 records remained. Screening of titles and abstracts yielded 49 records for full-text review. After reading the full texts, 28 studies were finally included. The detailed literature screening process is shown in Figure 1.

### 3.2 Quality assessment and basic characteristics of included studies

Following methodological quality assessment, among the 28 included studies (Xiang, 2005; Klavina and Block, 2008; Goudas and Magotsiou, 2009; Iserbyt et al., 2010; Cox and Ullrich-French, 2010; Iserbyt et al., 2011; Jõesaar et al., 2012; Li, 2013; Silva et al., 2014; Wang et al., 2018; Ingrell et al., 2019; Wang et al., 2022; Quan and Lu, 2020; Gao, 2013; Wang and Ye, 2021; Zhang, 2021; Zhao, 2021; Xu, 2021; Xia and Liu, 2022; Lee et al., 2022; Zhang, 2023; Ke, 2022; Mean and Kof, 2023; Li et al., 2024b; Romdhani et al., 2024; Ye et al., 2024; Wang, 2024; Guozhang et al., 2025). After synthesizing the ratings across all items, all 28 included studies were assessed as grade B. The basic characteristics of the included studies are presented in Table 1.The quality of the studies included in the review is shown in Table 2.

**Table 1:** Summary of included literature.

**References**	**Research institution**	**Research methods**	**Participants**	**Context**	**Phenomenon of interest**	**Main findings**
Xiang (2005)	Civil Aviation Flight University of China, Sports Dept.	Questionnaire, correlation analysis	Middle school students (*n* = 151)	Extracurricular sports	Impact of sports friendship quality on PA participation	1. Peers provide learning space for extracurricular sports learning 2. Sports friendship quality positively correlates with PA participation level 3. Age and gender differences exist in sports friendship quality
Klavina and Block (2008)	University of Latvia, etc.	Observation, experimental design	Elementary students (incl. severe multiple disabilities)	Inclusive PE class	Impact of peer tutoring on student interaction & activity time	1. Increased teaching & physical interaction under peer-mediated/voluntary support 2. Social interaction remained low 3. Student activity time increased, interaction with teacher decreased
Goudas and Magotsiou (2009)	University of Greece	Experimental (pre-post test)	6th graders (*n* = 114)	Cooperative PE curriculum	Impact of cooperative learning on social skills & group attitudes	Experimental group showed significant improvement in social skills & preference for group cooperation; cooperative learning promoted peer interaction
Iserbyt et al. (2010)	KU Leuven, etc.	Experimental, repeated measures ANOVA	Sports science students (*n* = 86)	Reciprocal peer tutoring (BLS skills)	Impact of role switching & definition on skill retention	1. Role switching & definition improved skill retention 2. High-guidance group performed better in retention phase
Cox and Ullrich-French (2010)	Washington State University	Cross-sectional survey, latent profile analysis	7th−8th graders (*n* = 244)	PE class	Influence of peer & teacher relationship characteristics on motivation	Identified three profiles: positive (high peer + high teacher support) had optimal motivation; Mixed (high peer + low teacher) still advantageous; Weak (low peer + low teacher) had poorest motivation
Iserbyt et al. (2011)	KU Leuven, etc.	Experimental, task cards & peer teaching	8th graders (*n* = 55)	Tennis skill teaching	Impact of peer teaching & task cards on motor skill acquisition	Peer teaching with task cards nearly as effective as teacher-centered model for technical skills (e.g., tennis), emphasizing dual achievement of social & motor goals
Jõesaar et al. (2012)	Estonian Sports University	Longitudinal, structural equation modeling	Adolescent athletes (*n* = 362)	Sports training environment	Relationship between coach autonomy support, peer motivational climate & intrinsic motivation	Coach autonomy support predicted subsequent task-involving peer climate; both jointly promoted intrinsic motivation
Li (2013)	Shanghai University of Sport	Experimental: peer education intervention	Urban children (*n* = 120)	School PE class	Impact of peer education on friendship quality & social adaptation	Peer education significantly improved primary school students' friendship quality and social adaptation skills
Silva et al. (2014)	American University	Structural equation modeling, accelerometry	High school students (*n* = 203)	Daily physical activity	Direct & indirect effects of parental & peer social support on MVPA	Both parental and peer support had direct effects on MVPA, and indirect effects via self-efficacy and enjoyment.
Wang et al. (2018)	China Institute of Sport Science	Mixed methods: questionnaire + interview	Junior High Students (*n* = 300)	PE class & extracurricular training	Impact of peer assistance on motor skill mastery	Peer assistance improved skill acquisition efficiency, enhanced learning motivation and cooperative awareness
Ingrell et al. (2019)	Swedish School of Sport and Health Sciences, etc.	Longitudinal, multilevel modeling	Adolescent student-athletes (*n* = 78)	Various sports training & competition	Influence of coach, peers, parents on achievement goal orientations	1. Task and ego orientation decreased with age 2. Task-involving peer climate positively correlated with task orientation 3. Ego-involving coach climate positively correlated with ego orientation
Wang et al. (2022)	Wuhan Sports University	Experimental: peer comparison intervention	Adolescents (*n* = 200)	Sports training camp	Impact of peer comparison on motivation & performance	Moderate peer comparison stimulated competitive awareness and improved sports performance
Quan and Lu (2020)	Northwestern Polytechnical University	OLS regression, coefficient bunching analysis	Junior high students (*n* = 7,591)	Daily physical exercise behavior	Strength of peer effects & gender differences	Peer effects strength was 80% of individual effect; boys more affected; opposite-sex friends significantly increased boys' exercise time
Gao (2013)	Beijing Sport University	Questionnaire, structural equation modeling	Adolescents (*n* = 800)	Extracurricular sports	Impact of peer support on physical exercise behavior	Peer support significantly positively predicted physical exercise frequency and intensity
Wang and Ye (2021)	Beijing Normal University	Questionnaire, path analysis	Middle school students (*n* = 500)	School sports activities	Relationship between peer pressure & physical exercise behavior	Positive peer pressure promoted exercise behavior; negative pressure inhibited participation intention
Zhang (2021)	Guangdong University of Finance	Questionnaire, nomination method	Adolescents (number not specified)	Daily physical exercise environment	Impact of peer acceptance on physical exercise behavior	Peer influence significant; number of friends, positive friends positively correlated with exercise time; boys more susceptible to peer influence
Zhao (2021)	Henan University	Questionnaire, mathematical statistics	Junior high students (*n* = 900)	Extracurricular sports activities	Impact of peer relationships on extracurricular sports participation	Positive peer relationships (affirmation/care, conflict resolution/help, intimacy/communication) significantly promoted PA intensity, time, frequency; Negative relationships (conflict/betrayal) negatively impacted most activities but positively incentivized competitive sports like basketball
Xu (2021)	Liaocheng University	Questionnaire, mathematical statistics	Shandong junior high students (*n* = 951)	In-school & extracurricular PA	Impact of peer effects on physical activity level	1. Peer PA level had strongest influence, peer attitude next, peer support weakest 2. Behavioral support most influential, verbal support next 3. Team interaction most influential, activity time next
Xia and Liu (2022)	Shanghai University of Sport	Literature review, theoretical analysis	Western sports research cases	Sports behavior & social interaction	Identification methods & application areas of peer effects	1. Peer effects widely exist in sports 2. Methods include SAR model, heterogeneity models, etc. 3. Applied to deviant behavior, social adaptation, health responses
Lee et al. (2022)	Sunchon National University, Sungkyul University, Korea	Cross-sectional survey, structural equation modeling	Multicultural adolescents (*n* = 192)	PA in multicultural contexts	Relationship between self-esteem, peer relationships, social withdrawal & multicultural acceptance	Self-esteem indirectly promoted multicultural acceptance via peer relationships; peer relationships mediated between self-esteem and acceptance; social withdrawal had no significant mediating effect
Zhang (2023)	East China Normal University	Qualitative: Grounded Theory	High school students (*n* = 25)	Sports club activities	Impact of peer relationships on persistence of sports participation	Peer acceptance and group belonging were key factors sustaining sports participation
Ke (2022)	Wuhan Sports University	Empirical analysis, questionnaire, regression, mediation analysis	Junior high students (*n* = 7,397)	Extracurricular & daily PA	Mechanism of sports participation & peer groups on adolescent deviant behavior	1. Sports participation significantly negatively influenced deviant behavior 2. PA time showed a “U-shaped” threshold effect 3. Positive peer groups partially mediated; negative peer groups partially/fully mediated 4. Significant differences in deviant behavior by gender, household registration, family background
Mean and Kof (2023)	Slovenian Sports Institute	Experimental (pre-post test, ANCOVA)	Adolescent athletes (*n* = 140)	Competitive athletics training	Cooperative learning vs. direct instruction on motor skills	Cooperative learning significantly superior to direct instruction on multiple motor skills, promoting peer teaching & feedback
Li et al. (2024b)	Guangzhou Sport University	Structural equation modeling, factor analysis	Adolescents (*n* = 6,067)	Daily PA & non-cognitive ability development	Mediating role of peer effects between PA & non-cognitive abilities	Peer effects partially mediated the relationship (effect share 24.5%); positive feedback promoted non-cognitive abilities
Romdhani et al. (2024)	Multinational (Tunisia, etc.)	Randomized crossover experiment	Sports science university students (*n* = 36)	CrossFit training environment	Impact of peer vs. coach verbal encouragement on performance & psychophysiological indicators	1. Peer encouragement superior to coach encouragement on 1-RM strength, endurance, RPE, FS 2. Peer encouragement more effective enhancing performance & psychological state
Ye et al. (2024)	China Agricultural University, Qujing Normal University	Mathematical statistics, panel data analysis	Migrant children (*n* = 2,288)	In-school sports participation	Peer effects in migrant children's sports participation	1. Peer effects showed an inverted U-shaped curve 2. Migration distance & duration influenced effect strength 3. Integration needs drive the effects
Wang (2024)	Huaibei Normal University	Qualitative: semi-structured interviews, observation	Junior high students (*n* = 30)	PE class, after-school, training venues	Influence of peer factors on specific motor skill formation	Four core categories: formative environment, motivational conditions, time foundation, interactive influence; peer relationships significantly promoted skill formation
Guozhang et al. (2025)	Southwest University, etc.	Two-way fixed effects, instrumental variables	Children/adolescents from multi-child families (*n* = 2,023)	Family environment PA behavior	Impact & mechanism of sibling PA on individual PA	Sibling PA significantly positively influenced individual exercise frequency (β = 0.085) & time (β = 0.208); mechanisms: PA propensity, sports support, exercise role models; Heterogeneity by gender, birth order, birth interval, athletic ability

**Table 2:** PEDro scores of included literature.

**References**	**Subjects were included in the conditions**	**Randomized**	**Assign hidden**	**The baseline issimilar**	**Subjects wereblinded**	**Participants wereblinded**	**Outcome measureswere blinded**	**More than 85% of people were given at least one primary outcome measure**	**Intention-to-treat analysis**	**Inter-group statistical report**	**Outcome point test values and variant measurements**	**PEDroscore**	**Quality evaluation**
Xiang (2005)	+	+	–	+	–	–	–	+	+	+	+	6	High quality
Klavina and Block (2008)	+	+	–	+	–	+	–	+	+	+	+	7	High quality
Goudas and Magotsiou (2009)	+	+	–	+	–	–	–	+	+	+	+	6	High quality
Iserbyt et al. (2010)	+	+	–	+	–	+	–	+	+	+	+	7	High quality
Cox and Ullrich-French (2010)	+	+	–	+	–	–	–	+	+	+	+	6	High quality
Iserbyt et al. (2011)	+	+	–	+	–	–	–	+	+	+	+	6	High quality
Jõesaar et al. (2012)	+	+	–	+	–	+	–	+	+	+	+	7	High quality
Li (2013)	+	+	–	+	–	+	–	+	+	+	+	7	High quality
Silva et al. (2014)	+	+	–	+	–	–	–	+	+	+	+	6	High quality
Wang et al. (2018)	+	+	–	+	–	+	–	+	+	+	+	7	High quality
Ingrell et al. (2019)	+	+	–	+	–	–	–	+	+	+	+	6	High quality
Wang et al. (2022)	+	+	–	+	–	–	–	+	+	+	+	6	High quality
Quan and Lu (2020)	+	+	–	+	–	—-	–	+	+	+	+	6	High quality
Gao (2013)	+	+	–	+	–	–	–	+	+	+	+	6	High quality
Wang and Ye (2021)	+	+	–	+	–	+	–	+	+	+	+	7	High quality
Zhang (2021)	+	+	–	+	–	+	–	+	+	+	+	7	High quality
Zhao (2021)	+	+	–	+	–	+	–	+	+	+	+	7	High quality
Xu (2021)	+	+	–	+	–	–	–	+	+	+	+	6	High quality
Xia and Liu (2022)	+	+	–	+	–	+	–	+	+	+	+	7	High quality
Lee et al. (2022)	+	+	–	+	–	–	–	+	+	+	+	6	High quality
Zhang (2023)	+	+	–	+	–	+	–	+	+	+	+	7	High quality
Ke (2022)	+	+	–	+	–	+	–	+	+	+	+	7	High quality
Mean and Kof (2023)	+	+	–	+	–	+	–	+	+	+	+	7	High quality
Li et al. (2024b)	+	+	–	+	–	–	–	+	+	+	+	6	High quality
Romdhani et al. (2024)	+	+	–	+	–	–	–	+	+	+	+	6	High quality
Ye et al. (2024)	+	+	–	+	–	+	–	+	+	+	+	7	High quality
Wang (2024)	+	+	–	+	–	+	–	+	+	+	+	7	High quality
Guozhang et al. (2025)	+	+	–	+	–	+	–	+	+	+	+	7	High quality

+ indicates that the corresponding indicator is met; – the representative does not meet the corresponding indicators; Baseline similarity refers to the similarity of groups at baseline for important prognostic measures; Blinding refers to blinding; More than 85% of people were measured for at least one primary outcome, meaning that if not reported, they were taken as negative outcomes; Intention-to-treat analysis means that all participants complete trials and tests as required, with at least one primary outcome if not, and negative outcomes if not reported.

### 3.3 Meta-synthesis results

Through repeated reading, comprehension, and analysis of the 28 included studies, 45 research findings were extracted. Findings with similar meanings were grouped into 12 categories, which were further synthesized into four integrated findings.

#### 3.3.1 Integrated finding 1: peer support and interaction—the foundational role of social relationships

##### 3.3.1.1 Category 1: emotional and behavioral support

Peers provide multi-dimensional support, laying a solid emotional and behavioral foundation for adolescents' participation in physical exercise. On the emotional level, companionship, encouragement, and affirmation from peers are key drivers for initiating and sustaining exercise behaviors [“My friends' cheers kept me going when I felt like stopping during long-distance running” (Silva et al., 2014)]. This positive emotional interaction effectively enhances individuals' exercise motivation and psychological resilience. On the behavioral level, immediate, concrete assistance and co-participation directly translate into higher exercise frequency and intensity [“Having classmates to play badminton with made me go to the court significantly more often each week” (Gao, 2013)]. Research further indicates that high-quality sports friendships, characterized by high trust, shared sports interests, and reliable mutual assistance, are significantly positively correlated with long-term participation in adolescent physical activity [“We are not just partners on the court; we also exchange tips and insights privately. This kind of friendship makes me love the sport even more” (Xu, 2021)]. Moreover, good peer relationships can effectively buffer feelings of frustration during exercise, providing a stable psychological support environment for sustained participation [“Even if we lose a match, teammates don't blame each other but summarize it together. This makes me dare to push myself next time” (Wang and Ye, 2021)].

##### 3.3.1.2 Category 2: team interaction and sense of belonging

Positive team interaction is a key contextual factor sustaining adolescent participation in physical exercise, while the resulting peer acceptance and group belonging are core drivers for long-term engagement. In team sports, shared goals and collaborative processes significantly enhance cohesion among members [“To win the class basketball game, we practiced tactics together after school. It felt like we were united, striving for the same goal” (Ingrell et al., 2019)]. This close interaction not only increases the enjoyment of the sport itself but also provides individuals with a strong sense of identity and value within the group [“Being a member of the school team makes me proud and demands higher standards from myself” (Zhang, 2023)]. Studies show that when adolescents feel accepted and needed within a sports team, their intrinsic motivation and willingness to persist in exercise significantly increase [“In the track and field team, no one looks down on others for running slowly. We encourage each other, making me feel this is my ‘home'. I want to stay even when it's tough” (Xu, 2021)]. Therefore, constructing a supportive and inclusive team environment is crucial for transforming external social attraction into adolescents' enduring internal drive for participation.

##### 3.3.1.3 Category 3: Conflict Resolution

Good peer relationships are not only the cornerstone of positive interactions but also possess the important function of effectively resolving conflicts and maintaining team stability, thereby providing a safe and stable social environment for adolescents' sustained participation in physical exercise. In team collaboration and competitive confrontations, disagreements, competitive friction, or blame for mistakes are inevitable. At such times, peer relationships built on mutual trust and respect can transform potential destructive conflicts into opportunities for deepening relationships through positive communication, perspective-taking, and timely forgiveness [“We almost argued over a bad pass during the game, but we talked it out right after, and now we understand each other's positioning habits better” (Zhao, 2021)]. This effective conflict management mechanism significantly reduces the risk of sports withdrawal or dropout due to interpersonal friction [“I wanted to quit before because I found a teammate too domineering, but after we all talked it out, now we get along and cooperate much more naturally” (Wang, 2024)]. Therefore, fostering adolescents' conflict resolution skills in sports contexts is a key link in maintaining team cohesion and ensuring the sustainability of their sports participation.

#### 3.3.2 Integrated finding 2: behavioral modeling and atmosphere—the driving role of group environment

##### 3.3.2.1 Category 4: behavioral modeling and imitation

In the social learning process of adolescent physical exercise, the role of peers as “behavioral models” exerts an irreplaceable influence. Social learning theory emphasizes that individuals acquire new behavioral patterns by observing and imitating others, a mechanism particularly evident in adolescent physical exercise (Xiang, 2005). First, peers' physical activity level becomes a key predictor of an individual's activity level. Multiple longitudinal studies confirm that adolescents unconsciously absorb their peers' exercise habits, gradually adjusting their own level of engagement (Quan and Lu, 2020). Second, the demonstration effect of peers plays a unique role in the acquisition of complex motor skills. Compared to traditional teacher demonstrations, peer demonstrations are more referential due to the proximity of ability levels. Motor skill learning theory suggests that learners are more inclined to imitate peers whose abilities are similar but slightly superior, a phenomenon Vygotsky termed the optimal learning space within the “zone of proximal development” (Iserbyt et al., 2011). In an experimental study on tennis forehand teaching, students in the peer demonstration group performed significantly better in skill retention tests than the control group receiving only teacher demonstration (Iserbyt et al., 2011). Furthermore, peer assistance creates high-quality informal learning environments. By observing peers' successful movements, analyzing their mistakes, and listening to their experience sharing, individuals can quickly form correct mental representations of movements and effectively avoid common errors (Wang et al., 2018). This “proximal demonstration” and immediate feedback mechanism based on peers significantly reduces the cognitive load of skill learning and accelerates the internalization and automatization process of motor skills. It is noteworthy that the effectiveness of peer modeling is moderated by various factors. Research shows that the model's ability level, the quality of the relationship with the observer, and the authenticity of the demonstration context all influence the effect of observational learning (Mean and Kof, 2023). When the model is perceived as a “similar other” and has a close relationship with the observer, their demonstration often elicits stronger motivation to imitate and more lasting behavioral change.

##### 3.3.2.2 Category 5: motivational climate shaping

In the social environment of adolescent physical exercise, the “motivational climate” collectively created by the peer group has a profound impact on individuals' participation motivation. Achievement goal theory distinguishes between two basic types: “task-involving” and “ego-involving.” Among these, the task-involving climate created by peers is confirmed to be a key socio-psychological factor in stimulating and maintaining adolescents' intrinsic motivation (Jõesaar et al., 2012). A task-involving climate is characterized by an emphasis on effort, improvement, cooperation, and skill mastery. In this climate, peers focus more on personal ability improvement than mere outcome comparison, and individuals perceive success as stemming from their own effort and progress, providing fertile ground for cultivating intrinsic motivation [“In our team, as long as you give your all on the court, even if we lose in the end, everyone will applaud you. We care more about whether we did better today than yesterday” (Ingrell et al., 2019)]. This collective value defining success as effort and progress effectively reduces fear of failure and enhances individuals' sense of autonomy and enjoyment in participation. In contrast, an ego-involving climate created by peers may undermine intrinsic motivation. When the peer group overemphasizes competition, social comparison, and an “outcome-only” focus (where only winning or being the best is recognized), it significantly increases psychological pressure and anxiety among adolescents. Especially for individuals with temporarily lower skill levels, this climate can easily lead to self-doubt and reduced interest in participation (Cox and Ullrich-French, 2010). A student in a qualitative interview admitted: “If the team only cares about who runs the fastest or scores the most, someone like me with a weaker foundation feels a lot of pressure, even afraid to train” (Wang and Ye, 2021). The task-involving climate promotes intrinsic motivation through several psychological mechanisms. First, by emphasizing effort and improvement, it helps individuals establish a controllable attribution for success, believing that they can improve their ability and succeed through their own efforts, which directly enhances their sense of behavioral autonomy (Jõesaar et al., 2012). Second, by encouraging cooperation and mutual assistance, it satisfies adolescents' need for belonging, providing them with stronger social connection and emotional support within the team (Ingrell et al., 2019). Third, by focusing on the learning process and personal progress, it effectively enhances the sense of skill mastery and enjoyable experience in sports (Mean and Kof, 2023).

##### 3.3.2.3 Category 6: peer comparison and competition

In the socialization process of adolescent physical exercise, peer comparison and competition constitute a complex and powerful influencing mechanism. Moderate social comparison can effectively stimulate competitive awareness and performance improvement. When adolescents observe the excellent performance or progress of peers, especially those of similar ability, it often triggers a competitive mindset of “not wanting to fall behind,” leading them to invest more effort to prove their own capability. An experimental study found that in a physical fitness test, the boys' group told to “compete against the class average” performed significantly better in the standing long jump than the control group instructed to “compete only against their own previous performance” (Wang et al., 2022). This moderate upward comparison can serve as an external driver in specific contexts, prompting individuals to step out of their comfort zone. However, the effects of peer comparison show significant gender differences. Multiple empirical studies indicate that male groups generally respond more positively to peer comparisons based on direct competition, and their sports performance shows greater improvement as a result (Quan and Lu, 2020; Zhang, 2021). This difference may be related to the differential socialization of gender roles early on, where society often encourages males more to demonstrate competence in competitive situations. A large-sample survey showed that, after controlling for other variables, having more opposite-sex peers (potentially triggering stronger competition and display motivation) had a significantly greater effect on increasing boys' physical exercise time than girls' (Quan and Lu, 2020). It is noteworthy that peer comparison has a clear “double-edged sword” effect. When comparison becomes excessive or the environment overemphasizes “winner-takes-all,” its positive effects quickly diminish and can even turn negative. For adolescents with lower skill levels or insufficient self-confidence, persistent unfavorable comparisons can easily induce anxiety, frustration, and “learned helplessness,” ultimately leading to decreased motivation for sports participation and withdrawal behavior (Wang and Ye, 2021). Therefore, the key lies in guiding adolescents toward constructive social comparison—focusing on peers' strategies and effort processes for learning, rather than merely comparing outcomes.

#### 3.3.3 Integrated finding 3: intrinsic motivation and identity—the mediating transformation of individual psychology

##### 3.3.3.1 Category 7: self-efficacy and enjoyment

In the psychological mechanisms through which peers influence adolescent physical exercise, self-efficacy and enjoyment of exercise serve as key mediating variables, translating external social support into individuals' enduring internal willingness and behavior. According to social cognitive theory, individual behavior is not only influenced by the environment but is also regulated by their beliefs about their own capabilities (i.e., self-efficacy), and peer support is an important source for shaping these beliefs. (1) Peer support is a key pathway to enhancing sports self-efficacy. When adolescents receive encouragement, positive feedback, or even concrete assistance from peers during physical exercise, they are more inclined to believe that they also possess the ability to complete specific sports tasks. A structural equation modeling study targeting high school students confirmed that peer support not only had a direct effect on physical activity level but also produced a significant indirect effect through self-efficacy (indirect effect value = 0.14, *p* < 0.01; Silva et al., 2014). A student who successfully completed their first 5-km run with peer encouragement shared: “Every time I wanted to give up, my friend would shout beside me, ‘You can do it, you're almost there.' Hearing those words, I really felt I could run a little further” (Xu, 2021). (2) Enjoyment of exercise is the internal lubricant sustaining participation behavior. Peer support and positive interaction can significantly enhance the inherent fun of the physical exercise process. This enjoyment stems not only from the dopamine release during exercise itself but also from the social pleasure and sense of belonging gained in shared activities. When physical exercise is closely associated with positive emotional experiences (such as the joy of cooperating with friends, the satisfaction of being accepted by the team), individuals' intrinsic motivation is greatly stimulated. Research indicates that in team sports, the quality of peer relationships can positively predict enjoyment of exercise, which in turn significantly influences future participation intention (β = 0.32, *p* < 0.001; Gao, 2013). (3) There is a synergistic enhancement effect between self-efficacy and enjoyment. On one hand, higher self-efficacy prompts individuals to be more willing to try and persist, thereby increasing opportunities for successful experiences and exercise enjoyment. On the other hand, strong enjoyment of exercise reinforces individuals' positive evaluation of their own abilities, further enhancing their self-efficacy. Together, these form a positive psychological feedback loop, allowing the long-term benefits of peer support to be sustained.

##### 3.3.3.2 Category 8: identity and integration needs

Among the psychological drivers of adolescent physical exercise, identity and the need for group integration constitute a deep-seated socio-psychological mechanism. This mechanism is particularly evident in specific groups (such as migrant children, transfer students, or those with weak foundational sports skills), becoming an important force driving their participation in physical exercise. (1) The need for group integration is the initial motivation triggering peer effects. For adolescents facing challenges in adapting to their social environment, participating in peer-led physical activities is an effective strategy for social integration. A panel study focusing on migrant children found that a strong need for group integration was a significant reason for the inverted U-shaped curve distribution of peer effects on their sports participation (β = 0.24, *p* < 0.01; Ye et al., 2024). In a qualitative interview, a junior high student who had moved to the city with their parents admitted: “When I first transferred schools, I didn't know anything. Going to play basketball with classmates was the quickest way to make friends. Even though my skills were poor at first, I kept going so I wouldn't be left out” (Zhao, 2021). This indicates that in this context, physical exercise transcends its singular function of fitness, becoming a social passport to group membership. (2) Peer relationships indirectly promote sports participation by influencing self-esteem levels. Social identity theory posits that individuals derive self-worth from their group membership. When adolescents are accepted and recognized within a sports group, their overall self-esteem level increases significantly. A study on multicultural adolescents confirmed that peer relationships fully mediated the relationship between self-esteem and group acceptance (effect size = 0.36, *p* < 0.001; Lee et al., 2022). This self-esteem, reinforced through peer relationships, subsequently translates into more positive attitudes toward sports participation and higher participation frequency. (3) The formation of identity exhibits dynamic developmental characteristics. As individuals deepen their participation in the sports group, their identity undergoes a transition from “instrumental identity” (participating to integrate) to “substantive identity” (participating out of passion). This transition process significantly enhances the stability and sustainability of sports participation. Research found that high school students who participated continuously in sports clubs for over a year had significantly higher levels of identity than short-term participants (*t* = 3.42, *p* < 0.01; Zhang, 2023).

##### 3.3.3.3 Category 9: non-cognitive ability development

In adolescent development research, non-cognitive abilities (such as perseverance, cooperative spirit, responsibility, and emotional regulation skills) have been proven to be important literacies influencing long-term individual development. This study found that peer effects play a key mediating role between physical exercise and the development of non-cognitive abilities, forming a self-reinforcing positive feedback loop. Physical exercise provides an authentic context for the development of non-cognitive abilities. Aspects such as rule adherence, coping with wins and losses, and cooperative coordination in team sports create a natural setting for cultivating perseverance and cooperative spirit. A structural equation modeling study involving 6,067 adolescents showed that peer effects partially mediated the relationship between physical exercise and non-cognitive abilities, with an effect share of 24.5% (Li et al., 2024b). This indicates that physical exercise not only directly promotes the development of non-cognitive abilities but also produces an indirect reinforcing effect through the social mechanism of peer interaction. On one hand, peer interaction is a key mechanism for cultivating specific non-cognitive abilities. In team sports situations, adolescents internalize character traits by observing specific behaviors of peers, such as persistence in the face of setbacks (“Seeing my teammate keep playing after getting injured taught me not to give up easily”) and tolerance during conflicts (“The captain never blames teammates for mistakes but encourages us to do better next time”). This process of observational learning and situational modeling makes abstract qualities like “perseverance” and “cooperation” concrete and learnable. On the other hand, non-cognitive abilities and peer effects form a bidirectional enhancement loop. Individuals with higher non-cognitive abilities find it easier to establish good peer relationships, thereby gaining more opportunities to participate in collective sports activities; rich peer sports interactions further promote the development of their non-cognitive abilities. This finding was verified in a longitudinal study: baseline cooperative ability significantly predicted peer acceptance 1 year later (β = 0.18), and peer acceptance in turn predicted the improvement of non-cognitive abilities (β = 0.26; Mean and Kof, 2023).

#### 3.3.4 Integrated finding 4: educational interventions and risk management—optimization strategies for practical application

##### 3.3.4.1 Category 10: structured intervention models

In the systematic application of peer effects to educational practice, structured intervention models demonstrate significant advantages. Numerous studies show that well-designed intervention strategies such as cooperative learning, peer tutoring, and peer education are more effective than traditional teacher-centered instructional models in enhancing students' motor skills, social abilities, and participation motivation. First, cooperative learning enhances comprehensive competencies by building positive interdependence. In this model, group members are assigned shared learning goals and clear role divisions, where each individual's success is closely linked to the team's overall performance. A physical education teaching experiment with sixth graders found that students in the cooperative learning situation showed significant improvement not only in social skills and preference for group cooperation (*p* < 0.01) but also mastered motor skills faster than the control group receiving traditional instruction (Goudas and Magotsiou, 2009). Second, peer tutoring creates a low-threat, high-feedback learning environment. Having peers of similar or slightly higher ability act as tutors can effectively reduce learners' anxiety and provide more timely and comprehensible movement corrections and psychological support. In a tennis skill teaching study, the group using task cards combined with peer teaching achieved skill acquisition effects almost as effective as the teacher-centered model, while better achieving the dual goals of social and motor skill development (Iserbyt et al., 2011). This supports the social constructivist view that knowledge (including motor skills) is constructed more effectively through collaboration and dialogue with peers. Third, peer education utilizes the power of role models to guide behavior and transmit values. When healthy, positive behavioral patterns are demonstrated and advocated by peers rather than authority figures, they are often more persuasive and influential. A “peer education” intervention experiment targeting urban primary school students confirmed that this model not only significantly improved students' sports friendship quality but also effectively enhanced their social adaptation skills (Li, 2013). This suggests that peer education can reach deep-seated attitudes and beliefs that are difficult to influence through traditional instruction. However, the key to the success of structured interventions lies in systematic design. Effective intervention is not simply about grouping students; it requires careful design of task structure, reward mechanisms, and individual accountability. For example, in reciprocal peer tutoring, clear role switching and role definition were proven crucial for improving skill retention (Iserbyt et al., 2010). These design elements work together to ensure the active engagement of every participant, avoid “free-riding,” and thus maximize the educational benefits of peer interaction.

##### 3.3.4.2 Category 11: risk identification and mitigation

While actively leveraging peer effects, it is essential to clearly identify and systematically mitigate their potential negative risks. Research shows that negative peer relationships and unbalanced social comparisons, if not effectively addressed, can significantly undermine adolescents' motivation for exercise and even harm their mental health. First, negative peer relationships, such as conflict, exclusion, or betrayal within a team, are significant risk factors leading to the discontinuation of adolescent sports participation. A survey of junior high school students showed that students who experienced peer conflict (e.g., being blamed by teammates, isolated within the team) had a 2.3 times higher risk of dropping out of sports activities in the subsequent month compared to those who did not (OR = 2.3, *p* < 0.01; Zhao, 2021). In a qualitative interview, a student who had quit the school basketball team admitted: “There were always a few people in the team who formed cliques. If you made a mistake, they would look at you with that kind of expression. Later, I felt too much pressure and didn't want to go anymore” (Wang, 2024). The psychological burden caused by such negative social experiences often outweighs the enjoyment derived from the sport itself. Second, over-reliance on peer comparison can turn into a harmful stressor. When the team atmosphere overemphasizes individual ranking and ability competition rather than effort and improvement, it can easily trigger anxiety and fear of failure, especially among adolescents with lower skill levels. A study on the motivational climate of physical education classes found that students in an ego-involving climate (overemphasis on winning and outperforming others) had significantly lower levels of intrinsic motivation than those in a task-involving climate (emphasis on effort and improvement; *t* = 4.32, *p* < 0.001; Cox and Ullrich-French, 2010). Third, group heterogeneity may lead to the marginalization of specific individuals. In mixed-ability groups, if the activity design and teacher guidance fail to fully consider individual differences, students with lower skill levels may be unintentionally excluded from the core interaction circle due to their inability to meet the group's skill requirements. An observational study on inclusive physical education classes found that although peer-mediated support increased the activity time of students with severe multiple disabilities, their level of social interaction remained low (Klavina and Block, 2008). This suggests that simply placing students together is insufficient; proactive strategies are needed to foster positive interactions. To effectively mitigate these risks, educational practice should focus on: (1) Strengthening monitoring and guidance of peer relationships, promptly identifying and mediating conflicts; (2) Guiding students to establish a healthy view of competition, emphasizing self-improvement over social comparison; (3) Adopting differentiated grouping strategies and providing multi-level task design to ensure that every student can find their place and value in the group.

##### 3.3.4.3 Category 12: heterogeneity and differentiated guidance

The effects of peer influence are not uniform; they exhibit significant heterogeneity across different groups and contexts. Therefore, educational interventions must adopt differentiated guidance strategies based on individual characteristics and specific situations to maximize positive effects while avoiding potential risks. First, the strength of peer effects shows clear gender differences. Multiple studies consistently indicate that male adolescents are generally more susceptible to peer influence in physical exercise. A large-sample regression analysis showed that the peer effects coefficient for boys (β = 0.62) was significantly higher than that for girls (β = 0.41), and the presence of opposite-sex friends had a significantly greater effect on increasing boys' exercise time than girls' (Quan and Lu, 2020). This suggests that in practical applications, interventions for boys could more effectively leverage peer modeling and competitive mechanisms, while for girls, more emphasis might be placed on cooperative and supportive peer relationships. Second, the influence of peer effects varies across different family backgrounds. A study on migrant children found that the strength of peer effects on their sports participation showed an inverted U-shaped curve, influenced by migration distance and duration (Ye et al., 2024). This indicates that for groups with a strong need for social integration, peer effects can serve as a powerful lever for promoting sports participation. Similarly, research on children from multi-child families revealed that sibling physical activity level significantly influenced individual exercise frequency (β = 0.085) and time (β = 0.208), with notable heterogeneity by gender, birth order, birth interval, and athletic ability (Guozhang et al., 2025). This suggests that family-based peer interventions should also consider these structural factors. Third, the effectiveness of peer effects is moderated by individual personality traits and social status. Students with higher self-esteem or social competence can better leverage the positive aspects of peer relationships, while those with lower self-esteem or social anxiety may be more vulnerable to negative peer influences. A study on multicultural adolescents found that self-esteem indirectly promoted multicultural acceptance through peer relationships, but this mediating effect was not significant for students with high social withdrawal tendencies (Lee et al., 2022). This reminds us that for students with specific personality traits, direct peer interventions may not be sufficient, and additional psychological support may be needed. Therefore, differentiated guidance strategies should include: (1) Gender-specific intervention approaches, designing peer activity content that aligns with the social interaction preferences of different genders; (2) Family background considerations, tailoring peer support systems to the specific needs of groups like migrant children; (3) Personality trait matching, providing additional social skill training for students with social interaction difficulties to help them better integrate into peer groups.

## 4 Discussion

### 4.1 The multi-dimensional nature of peer effects in adolescent physical exercise

This study reveals through meta-synthesis that peer effects on adolescent physical exercise are multi-dimensional, encompassing emotional, behavioral, and cognitive aspects, and their mechanisms are complex and interactive. This finding aligns with the core concepts of social cognitive theory, which emphasizes the dynamic interaction between individual behavior, environmental factors, and personal cognition. In the context of adolescent physical exercise, peers constitute a crucial micro-environmental factor, while individual psychological variables such as self-efficacy, enjoyment, and identity mediate the transformation of external social influence into internal behavioral motivation.

First, the emotional support dimension of peer effects is foundational. The companionship, encouragement, and affirmation provided by peers meet adolescents' basic psychological needs for belonging and security, creating favorable emotional conditions for initiating and sustaining exercise behaviors. This finding is consistent with the conclusion of self-determination theory that relatedness is one of the three basic psychological needs. When adolescents feel cared for and supported by peers in physical exercise, their intrinsic motivation is more easily stimulated, and the behavior is more likely to be internalized and autonomously regulated.

Second, the behavioral modeling dimension of peer effects is direct and referential. Through observation, imitation, and learning from peers, adolescents can quickly acquire motor skills and behavioral patterns, a process that aligns with the “vicarious learning” mechanism in social learning theory. The proximity of peer ability levels makes their demonstrations more relatable and achievable, creating an optimal learning space within the “zone of proximal development.” This explains why peer-assisted learning is often more effective than traditional teacher-centered instruction in certain contexts.

Third, the motivational climate dimension of peer effects is profound and enduring. The values and behavioral norms collectively shaped by the peer group subtly influence individuals' definitions of success and perceptions of ability. A task-involving climate that emphasizes effort, improvement, and cooperation helps cultivate healthy achievement goals and intrinsic motivation, whereas an ego-involving climate that overemphasizes competition and social comparison may undermine the participation motivation of some adolescents. This echoes the core viewpoint of achievement goal theory regarding the impact of motivational climate on individual behavior.

### 4.2 The dual nature of peer effects: positive promotion and potential risks

This study further reveals the dual nature of peer effects, which can both positively promote adolescent physical exercise and potentially bring certain risks. This dual characteristic requires special attention in educational practice.

On the positive side, peer effects can significantly enhance adolescents' participation frequency, skill level, and persistence in physical exercise through multiple pathways. Peer support provides emotional security, behavioral modeling offers learning references, and positive motivational climate shapes healthy values. These positive effects are not isolated but interact and reinforce each other, collectively promoting the development of adolescent physical exercise behavior. For example, peer support enhances self-efficacy, which in turn strengthens enjoyment of exercise, forming a virtuous cycle that sustains participation behavior.

However, potential risks of peer effects cannot be ignored. Over-reliance on peer relationships may lead to dependency, where once peer support is absent, individuals' exercise behavior may be interrupted. Negative peer relationships, such as conflict and exclusion, can directly lead to sports dropout. Unhealthy peer comparisons and excessive competitive pressure may trigger anxiety and reduce motivation for participation. Additionally, group heterogeneity may marginalize specific individuals, preventing them from benefiting from peer interactions.

This dual nature suggests that in educational practice, we should not only actively leverage the positive aspects of peer effects but also remain vigilant to their potential risks, implementing targeted preventive and intervening measures.

### 4.3 Practical implications and future research directions

Based on the above findings, this study proposes the following practical implications and future research directions:

First, educational practice should focus on constructing scientific peer support systems. For instance, research findings support structured intervention models such as “cooperative learning” or “peer tutoring” (e.g., Goudas and Magotsiou, 2009; Iserbyt et al., 2011). In practice, teachers can establish clear shared objectives and role definitions for groups, and design task cards to guide peers in providing skill feedback, thereby maximizing the positive effects of peer interaction.

Second, educational practice needs to strengthen risk management of peer effects. This includes promptly identifying and mediating negative peer relationships, guiding students to establish healthy competitive attitudes, and avoiding the negative impact of excessive social comparison. For students with specific characteristics (such as those with weak foundational skills or social interaction difficulties), additional support and guidance should be provided to help them integrate into peer groups.

Third, future research should further explore the mechanisms of peer effects in different cultural contexts. Most current studies are based on Western cultural backgrounds, and research within Eastern cultural contexts, especially the Chinese educational environment, remains insufficient. Future research could delve into how cultural values (such as collectivism vs. individualism) moderate the manifestation and effects of peer influence.

Fourth, future research could adopt more rigorous experimental designs and long-term tracking studies to reveal the long-term effects and dynamic development processes of peer effects. Most current studies are cross-sectional, limiting causal inference. Future research could use randomized controlled trials or long-term cohort studies to more accurately assess the causal effects of peer interventions and their lasting impacts.

### 4.4 Improve the educational intervention system to achieve precise guidance and risk prevention and control of peer effects

Educational intervention is a key link in translating research findings on peer effects into practical outcomes. This study finds that scientific and systematic educational interventions can not only effectively leverage the positive aspects of peer effects but also significantly reduce their potential risks, providing an important guarantee for the healthy development of adolescent physical exercise.

In terms of intervention model innovation, structured design has been proven to be the core element for enhancing intervention effectiveness. Intervention strategies based on peer interaction, such as cooperative learning, peer tutoring, and peer education, maximize educational benefits through carefully designed task structures, role assignments, and evaluation mechanisms. Specifically, cooperative learning prompts group members to work together to achieve common goals by building positive interdependence; peer tutoring utilizes the demonstration effect of similarly capable peers to create a low-threat, high-feedback learning environment; peer education effectively transmits healthy behaviors and values through the influence of role models. The key to the success of these intervention models lies in their systematic design—clear task objectives, well-defined individual responsibilities, appropriate difficulty gradients, and timely feedback mechanisms collectively ensure the deep engagement and effective development of every participant.

In terms of risk identification and control, this study reveals three potential risks associated with peer effects: participation interruption due to negative peer relationships, psychological pressure caused by excessive social comparison, and the weakening of intrinsic motivation due to peer dependency. If these risks are not addressed promptly, they can not only negate the positive impact of peer effects but may also adversely affect adolescents' mental health and the development of long-term exercise habits. Empirical data show that students who have experienced peer conflict have a significantly increased risk of dropping out of physical activity (OR = 2.3), and in highly competitive environments, approximately 34% of adolescents develop exercise avoidance tendencies due to fear of poor performance. These findings warn us that corresponding risk warning and intervention mechanisms must be established while utilizing peer effects.

In terms of differentiated application strategies, precise interventions based on group characteristics are particularly important. This study found that the influence of peer effects varies significantly by gender, age, and cultural background. Boys generally respond more positively to competitive feedback and attention from opposite-sex peers, while girls place greater importance on emotional support and a cooperative atmosphere; younger students are more easily influenced by direct encouragement, while older students are more influenced by the subcultural norms of their peer groups; special groups, such as migrant children, show unique sensitivity to peer behavior patterns. These differences require educational interventions to abandon the “one-size-fits-all” approach and instead adopt more refined, stratified, and categorized guidance strategies.

## 5 Conclusion

This study employed a meta-synthesis approach to systematically integrate research on peer effects in adolescent physical exercise, forming four integrated findings: Peer Support and Interaction; Behavioral Modeling and Atmosphere; Intrinsic Motivation and Identity; and Educational Interventions and Risk Management. The results indicate that peer effects influence adolescent physical exercise through multiple pathways, including emotional support, behavioral modeling, and motivational climate, demonstrating multidimensionality and complexity. Compared to previous studies, this review is the first to systematically reveal its multidimensional interactive mechanisms through meta-synthesis and explicitly propose a “benefit-risk” dual framework, providing a more systematic theoretical perspective and practical pathway for promoting adolescent physical exercise. At the same time, the limitations of this study should be noted: The methodological quality of the included literature varies, which may affect the strength of the evidence; most studies are based on Western or urban Chinese contexts, and the generalizability of the conclusions across different cultural and socio-economic environments requires further verification; moreover, the study protocol was not prospectively registered on platforms such as PROSPERO. Additionally, existing research is predominantly cross-sectional and short-term, and the long-term effects and dynamic mechanisms still need to be clarified through more longitudinal studies. Future educational practices should focus on constructing scientific peer support systems while emphasizing differentiated implementation and risk monitoring to promote the sustainable and healthy development of adolescent physical exercise behaviors.

## Data Availability

The original contributions presented in the study are included in the article/supplementary material, further inquiries can be directed to the corresponding author.
